# Human Illnesses and Animal Deaths Associated with Freshwater Harmful Algal Blooms—Kansas

**DOI:** 10.3390/toxins7020353

**Published:** 2015-01-30

**Authors:** Ingrid Trevino-Garrison, Jamie DeMent, Farah S. Ahmed, Patricia Haines-Lieber, Thomas Langer, Henri Ménager, Janet Neff, Deon van der Merwe, Edward Carney

**Affiliations:** 1 Kansas Department of Health and Environment, 1000 SW Jackson Street, Suite 075, Topeka, KS 66612, USA; E-Mails: fahmed@kdheks.gov (F.S.A.); PHaines-Lieber@kdheks.gov (P.H.-L.); tlanger@kdheks.gov (T.L.); hmenager@kdheks.gov (H.M.); jneff@kdheks.gov (J.N.); CEdwardCarney@aol.com (E.C.); 2 Florida Department of Health, 4052 Bald Cypress Way, Tallahassee, FL 32399, USA; E-Mail: Jamie.DeMent@flhealth.gov; 3 Department of Diagnostic Medicine/Pathobiology, College of Veterinary Medicine, Kansas State University, 1800 Denison Avenue, Manhattan, KS 66506, USA; E-Mail: dmerwe@vet.k-state.edu

**Keywords:** blue-green algae, canine, cyanobacteria, cyanotoxin, dog, harmful algal bloom, hepatotoxin, microcystin

## Abstract

Freshwater harmful algal bloom (FHAB) toxins can cause morbidity and mortality in both humans and animals, and the incidence of FHABs in the United States and Kansas has increased. In 2010, the Kansas Department of Health and Environment (KDHE) developed a FHAB policy and response plan. We describe the epidemiology of FHAB-associated morbidity and mortality in humans and animals in Kansas. Healthcare providers and veterinarians voluntarily reported FHAB-associated cases to KDHE. An investigation was initiated for each report to determine the source of exposure and to initiate public health mitigation actions. There were 38 water bodies with a confirmed FHAB in 2011. There were 34 reports of human and animal FHAB-associated health events in 2011, which included five dog deaths and hospitalization of two human case patients. Five confirmed human illnesses, two dog illnesses and five dog deaths were associated with one lake. Four human and seven dog cases were exposed to the lake after a public health alert was issued. Public health officials and FHAB partners must ensure continued awareness of the risks to the public, educate healthcare providers and veterinarians on FHAB-related health events and encourage timely reporting to public health authorities.

## 1. Introduction

Cyanobacteria, also known as blue-green algae, are found throughout the world in a variety of aquatic environments. This ancient class of microorganisms includes multiple species that produce some of the most powerful toxins known to man [1]. When environmental conditions are favorable, cyanobacteria can proliferate to dominate the phytoplankton within a body of water and form a bloom [2]. The cyanobacteria within the bloom can produce toxins that adversely affect human and animal health. The majority of freshwater cyanobacteria toxins are classified into two categories; hepatotoxins (toxins that target the liver) and neurotoxins (toxins that target the nervous system) [3]. Microcystin, a hepatotoxin, is produced by multiple species of cyanobacteria within the genera *Microcystis*, *Planktothrix*, *Anabaena* and *Oscillatoria* [3]. Microcystins are the most frequently occurring and widespread cyanotoxin throughout the world and the most commonly found cyanotoxin in Kansas lakes [4,5]. The effects of microcystin poisoning depend on the route of exposure (e.g., ingestion, inhalation, direct contact) and the amount of toxin to which the human, or animal, has been exposed. The onset of signs and symptoms can occur within minutes to hours of exposure. Clinical signs and symptoms of acute microcystin poisoning, in both animals and humans, are non-specific and can include; nausea, vomiting, diarrhea, cough, sore throat, rash and liver damage [4,6,7,8]. Most people with recreational water exposure to cyanobacteria recover without sequelae; however, the outcome for most dogs is death [4,6,7,8].

The incidence of freshwater harmful algal blooms (FHABs) has increased over the last three decades. In the United States, there were three FHAB-associated outbreaks from 1978 to 2008 compared to 11 outbreaks from 2009 to 2010 reported to the Waterborne Disease Outbreak Surveillance System (WBDOSS) and the Harmful Algal Bloom-Related Illness Surveillance System (HABISS) [8]. An outbreak must meet the following criteria; two or more people linked epidemiologically and the epidemiologic evidence must implicate recreational water as the probable source of illness [8]. Ten states (Florida, Iowa, Maryland, Massachusetts, New York, Oregon, South Carolina, Virginia, Washington, and Wisconsin) received grants to participate in HABISS; however, any state could report FHAB-associated outbreaks to the system. Although HABISS has been discontinued, voluntary reports of FHAB-associated outbreaks can be made to WBDOSS through the National Outbreak Reporting System (NORS) [8]. Anecdotal case reports and case studies have provided information on a wide range of acute illnesses associated with recreational exposure to cyanobacteria and their toxins; however, there is limited availability of epidemiological data [2].

By 2010, the Kansas Department of Health and Environment had received reports of and tested water bodies for FHABs for more than 25 years. However, there was no formal policy in place to protect public health. No U.S. Federal policy, regulations or guidelines exist for FHABs in recreational waters, although several states have developed their own policies [1,9,10]. In 2010, the Kansas Department of Health and Environment established a policy and response plan for FHABs in public waters. The public health Advisory and Warning levels were based on World Health Organization recommendations for recreational water use [4]. The policy included an active response to reports of human or animal illness or death potentially associated with a FHAB. The response included a case investigation and collection of water samples from the implicated body of water. Here, we describe the epidemiology of human and animal morbidity and mortality associated with freshwater harmful algal blooms in Kansas.

## 2. Results

### 2.1. FHAB Identification and Water Sampling

Public water bodies were sampled for FHAB when: (1) a report of a FHAB in a public body of water was received, or (2) a suspected FHAB-related illness in animals or humans was identified and reported to KDHE. A public water body was defined as those waters referred to as reservoirs, community lakes, state fishing lakes or were waters managed or owned by federal, state, county or municipal authorities. Also included were all privately-owned lakes that served as a public drinking water supply or were open to the general public for primary or secondary contact recreation [11]. In 2011, there were 42 water bodies reported to KDHE with a suspect harmful algal bloom; 38 water bodies were confirmed with a FHAB. A report of FHAB-associated human illness, Case 1, triggered sampling of one water body.

Between March 18 and October 31, 2011, 16 water bodies met the criteria for the Warning level (≥100,000 cyanobacteria cells/mL, ≥20 µg/L microcystin toxin or visible cyanobacteria surface accumulation) and four water bodies met the Advisory level (20,000 to <100,000 cyanobacteria cells/mL or detectable to <20 μg/L microcystin toxin) classification for at least one week due to a high cell concentration, elevated microcystin toxin or a combination of both.

We report the water sample results for Milford Lake, the largest man-made lake in Kansas, with 15,700 surface acres of water and 163 miles of shoreline [12]. Five locations were sampled at Milford Lake weekly beginning July 18 through October 10, 2011, for a total of 55 samples. We report the maximum, minimum, median and mean for cyanobacteria cell concentration (Table 1) and microcystin toxin level each week (Table 2). Milford Lake was issued a public health alert for FHAB conditions for 12 consecutive weeks (July 18–October 13, 2011). The highest maximum cyanobacteria cell concentration (5,576,000/mL) and microcystin toxin level (1,600 μg/L) occurred in samples taken August 22, 2011. The maximum total cyanobacteria cell concentration corresponded to the maximum microcystin enzyme-linked immunosorbent assay (ELISA) in all samples; however, the minimum cell concentration did not correspond to the minimum microcystin ELISA in two samples taken on August 29 and September 19. The predominant cyanobacteria type was *Microcystis* spp. in 82% (45/55) of the samples. *Anabaena* spp. was the predominant cyanobacteria type in four samples taken on or after September 26.

**Table 1:** Milford Lake cyanobacteria cell concentration, 2011 *.

Date of Collection (2011)	Maximum Total Cyanobacteria Cell Concentration * (No./mL)	Minimum Total Cyanobacteria Cell Concentration * (No./mL)	Median Cyanobacteria Cell Concentration * (No./mL)	Mean Cyanobacteria Cell Concentration * (No./mL)
July 18	1,825,000	6000	104,000	458,000
July 25	98,000	3000	28,000	28,000
August 1	335,000	31,000	252,000	189,000
August 8	60,000	4000	60,000	43,000
August 22	5,576,000	132,000	830,000	1,536,000
August 29	171,000	13,000	31,000	56,000
September 6	1,096,000	176,000	231,000	388,000
September 12	1,530,000	159,000	414,000	577,000
September 19	19,000	2,000	7000	9000
September 26	560,000	0	11,000	117,000
October 10	2000	0	300	700

* The data presented are a composite of all five sample sites from the same date.

**Table 2:** Milford Lake microcystin toxin level, 2011 *.

Date of Collection (2011)	Maximum ELISA * (µg/L)	Minimum ELISA * (µg/L)	Median ELISA * (µg/L)	Mean ELISA * (µg/L)
July 18	110	1	9	30
July 25	60	<1	3	15
August 1	60	6	35	30
August 8	9	6	6	7
August 22	1600	25	250	441
August 29	150	15	15	38
September 6	20	5	12	13
September 12	1000	12	180	322
September 19	6	2	2	3
September 26	60	0	2	13
October 10	0	0	0	<1

* The data presented are a composite of all five sample sites from the same date.

### 2.2. Trend Analysis

We evaluated water sample data collected from 1989 to 2009 and assigned a public health alert to each water body based on the criteria set forth in the KDHE 2010 FHAB Policy and Response Plan. Between 1989 and 2009, there were 413 water bodies that met the public health alert criteria (274 advisories and 139 warnings) [13]. We compared the median number of public health alerts that would have been issued based on the 2010 FHAB alert levels. We grouped the data into seven-year increments (1989–1995, 1996–2002 and 2003–2009). The median number of FHABs increased between each increment; 13 (1989–1995), 18 (1996–2002) and 25 (2003–2009). KDHE did not receive reports of human or animals cases associated with FHABs prior to 2011; however, a review of canine cyanotoxin poisonings in the United States by Backer *et al*., found two media reports of FHAB-associated deaths of four dogs in Kansas in 2007 and 2008 [6]. Veterinarians play a key role in the detection of FHABs, as they may be the first healthcare provider to recognize signs of FHAB-illness in animals [6]. Timely reporting of FHABs to public health officials is crucial to prevent human morbidity and additional animal mortality.

### 2.3. Outreach to Physicians and Veterinarians

Harmful algal bloom-related illnesses in humans are not considered reportable conditions in Kansas. However, any outbreak of any disease or condition is required to be reported to KDHE per Kansas statute. Similarly, FHAB-related illnesses and deaths among animals are not required to be reported to the Kansas Department of Agriculture.

Human and animal cases could be reported via phone call to KDHE or through the KDHE Harmful Algal Bloom website (http://www.kdheks.gov/algae-illness/index.htm). No cases of human or animal illness were reported in 2010. During 2011, KDHE sent letters to physicians and veterinarians to increase awareness of FHAB-related illness and encourage reporting of cases. A survey was administered to veterinarians and physicians during 2012 to evaluate the effectiveness of the messaging campaign and knowledge of public health advisories and warnings in or near their area of practice [14]. Although respondents displayed an increase in awareness of the adverse health effects caused by exposure to FHABs from 2010 to 2011, the majority were not aware of FHABs that had occurred in their county of practice or the surrounding counties [14]. KDHE and all FHAB stakeholders must continue to educate both human and animal healthcare providers regarding diagnosis and reporting of FHAB-related illnesses and deaths throughout the FHAB season each year.

### 2.4. Human Cases

KDHE received 25 reports of human illnesses potentially associated with FHABs in 2011. Three reports were anonymous complaints, and we were unable to complete an investigation. Of the 25 reports, nine were classified as not a case, primarily due to the healthcare provider failing to rule out other potential causes of illness. The remaining 13 cases were classified as suspect (*n* = 1), probable (*n* = 5) and confirmed (*n* = 7). Of the seven confirmed human illnesses, the median age was 40 years (range: 17–63 years); 71% (5/7) were male, and 29% (2/7) were hospitalized (Table 3). All human cases survived. Primary symptoms included: 71% (5/7) eye and upper respiratory tract irritation, 29% (2/7) rash and 14% (1/7) gastrointestinal. The most common primary route of exposure included direct contact, 100% (7/7), followed by ingestion, 43% (3/7), and inhalation, 14% (1/7). The median time from exposure to symptom onset was 24 h, with a range of 3–48 h.

Case 2, a 17-year-old male, presented to an emergency department on July 23, 2011. His symptoms included; sore throat, cough, malaise, headache and a fever of 104.1 °F. He did not report a rash. His symptoms began approximately 24 h after swimming at Milford Lake on July 20, 2011. He was diagnosed with pneumonia and hospitalized for three days for supportive care. A report of a potential FHAB at Milford Lake was reported to KDHE on July 18, 2011, and five water samples from high-traffic public access points were taken the same day. The water sample testing confirmed high cyanobacteria cell concentrations and microcystin toxin levels at a public health Warning level, and a press release was issued on July 22, 2011.

**Table 3:** Confirmed human freshwater harmful algal bloom-associated cases, Kansas, 2011.

Case	Sex	Age (Years)	Exposure Date	Time to Symptom Onset (h)	Primary Route of Exposure	Primary Symptoms	Recreational Water Activity
1	Male	63	06/15/11	7.5	Direct Contact	Rash	Fishing
2 *	Male	17	07/20/11	24	Direct Contact, Ingestion	Eye and Upper Respiratory Tract Irritation	Swimming
3	Female	52	07/21/11	48	Direct Contact	Eye and Upper Respiratory Tract Irritation	Swimming
4	Female	42	07/26/11	24	Direct Contact, Ingestion, Inhalation	Eye and Upper Respiratory Tract Irritation	Knee Boarding
5	Male	38	07/30/11	48	Direct Contact	Eye and Upper Respiratory Tract Irritation	Swimming
6 *	Male	38	08/05/11	3	Direct Contact, Ingestion	Eye and Upper Respiratory Tract Irritation and Rash	Water Skiing
7	Male	40	08/12/11	24	Direct Contact, Ingestion	Gastrointestinal	Jet Skiing

* Case 2 and Case 6 were hospitalized.

Case 6, a 38-year-old male, presented to an emergency department on August 8, 2011, with symptoms that included headache, joint pain, fatigue, sore throat, fever (102.5 °F), chills and diaphoresis. Healthcare providers initially suspected meningitis, but this, and other infectious diseases, were ruled out. He reported swallowing water when he fell in the lake while water skiing at Milford Lake on August 5, 2011. Milford Lake was under a public health Warning due to high cyanobacteria cell concentrations and microcystin toxin levels. He was diagnosed with cyanobacteria toxicosis. He was hospitalized for three days for supportive care.

In 2012, KDHE investigated five probable and two suspected cases. In 2013, there was one suspected case of FHAB-related morbidity in humans.

### 2.5. Canine Cases

In 2011, there were seven reports of FHAB-associated illnesses and deaths in dogs: one suspected, one confirmed illness and fiver confirmed deaths. One dog illness was classified as not a case. Of the six confirmed dog illnesses and deaths, the median age was 1.3 years (range: four months–six years), and 50% (3/6) were male (Table 4); the median weight was 51 pounds (range: 40–60 pounds). The median time from exposure to onset of clinical signs was 3.5 h (range: 1–48 h). Clinical signs included vomiting, diarrhea, lethargy, staggering, seizures and death.

**Table 4:** Confirmed canine freshwater harmful algal bloom-associated cases, Kansas, 2011.

Case	Sex	Age	Breed	Date of Exposure	Time to Onset of Signs (h)	Route of Exposure	Initial Signs	Outcome	Maximum ALT Level (μ/L); Ref. Range: 10–109 μ/L	Toxicology	Pathology
1 ^†^	Female	7 months	German Shepherd	08/12/11	48	Direct Contact, Ingestion, Inhalation	Lethargy	Died 08/16/11	>6,000	N/A	N/A
2 ^†^	Male	4 months	German Shepherd	08/12/11	48	Direct Contact, Ingestion	Vomiting	Survived	5,889	N/A	N/A
3 ^‡^	Male	3 years	Vizsla	08/17/11	3	Direct Contact, Ingestion	Vomiting Lethargy	Died 08/18/11	21,916	N/A	Hepatocellular necrosis, massive, acute and renal tubular epithelial necrosis, diffuse severe
4 ^‡^	Male	5 months	Vizsla	08/17/11	3	Direct Contact, Ingestion	Vomiting Lethargy	Died 08/18/11	3,378	*Microcystis* spp. on hair.	Hepatocellular necrosis, massive, acute
5	Female	2 years	Weimaraner	08/25/11	1	Direct Contact, Ingestion	Vomiting Lethargy Weakness	Survived	60,585	No cyanobacteria on hair	N/A
6	Female	6 years	Briard	09/24/11	4	Ingestion	Vomiting	Died 09/26/11	39,326	*Microcystis *spp. in vomitus	Hepatocellular necrosis, massive, acute and renal proximal tubular necrosis, acute

^†^ Case 1 and 2 from the same household; ^‡^ Case 3 and 4 from the same household.

All six confirmed canine cases had at least one alanine aminotransferase (ALT) test performed (Table 4). ALT is a serum liver enzyme biomarker that can be measured to determine the presence of injury to the liver. The largest increases in ALT develop with hepatocellular necrosis and inflammation [15]. The highest ALT level is reported for those dogs with multiple tests. The median ALT (13,958 μ/L) was higher than the reference range of ALT for dogs (10–109 μ/L).

Cases 3, 4, 5 and 6 were treated at the Kansas State University Animal Health Center in Manhattan, Kansas. *Microcystis* spp. was identified on hair samples from Case 4 and in vomitus from Case 6 [16]. No cyanobacteria were found on hair from Case 5; however, the dog was bathed prior to testing of the hair [16]. The stomach contents of Case 4 were examined for the presence of cyanobacteria; the dog vomited numerous times prior to death; therefore, no cyanobacteria were identified. A necropsy was performed on Cases 3, 4, and 6. All three dogs had massive, diffuse, acute hepatic necrosis consistent with microcystin toxicity. Case 5 developed liver failure and had the highest ALT level (60,585 μ/L); this dog survived with intensive supportive care [17].

In 2012, there was one suspected case, and in 2013, there were two suspected cases of FHAB-related morbidity reported to KDHE in dogs. There was one canine FHAB-associated fatality due to exposure to Milford Lake in 2013.

Recently, a case report was published by Rankin *et al*., who described the use of oral cholestyramine to successfully treat a dog with acute cyanobacterial toxicosis [18]. Cholestyramine is a bile acid sequestrant; it adsorbs and combines with bile acids in the intestine, where it forms an insoluble complex that is excreted in the feces [19]. This prevents enterohepatic recirculation of bile acids and any associated bound substances, such as microcystin toxins [18]. In addition, cholestyramine has been used to treat human patients with possible estuary-associated syndrome (PEAS), caused by an estuarine dinoflagellate that produces a neurotoxin [20]. Veterinarians and physicians should consider the use of cholestyramine, in addition to supportive care, for patients with suspected cyanobacterial toxicosis.

## 3. Experimental Section

### 3.1. FHAB Identification and Water Sampling

Water samples were collected in accordance with the KDHE Bureau of Environmental Field Service’s Lake Monitoring Protocol [21]. This protocol has been in place for the last thirty years to standardize monitoring of lakes to protect public health. Sample locations included areas identified as the most frequently used points of public access, such as swimming beaches, boat docks and ramps, marinas and public drinking water intakes. Water samples were collected by trained field staff using a beaker on a telescopic pole. The beaker was submerged approximately 1–2 inches below the water’s surface. Two collections were made, and the contents of the beaker were transferred to a one-liter cubitainer. The cubitainer was marked with the sample location identification number, collection time, collection date and the collector’s name. The cubitainer was placed in a cooler with ice to keep the sample cool, but not frozen. All samples were transported to the Kansas Health and Environmental Laboratories within twenty-four hours. Analysis of the samples included cyanobacteria cell concentrations and microcystin toxin analysis via enzyme-linked immunosorbent assay (ELISA). A public health Advisory was issued if the water test results had cyanobacteria cell concentrations of 20,000 to <100,000 cells/mL or microcystin toxin that was detectable to <20 µg/L. The water body would be re-tested within 4 weeks. The Advisory remained in effect until cyanobacteria concentrations were <20,000 cyanobacteria cells/mL and microcystin toxin concentrations were no longer detectable at all sample sites. A public health Warning was issued if the water test results had cyanobacteria cell concentrations ≥100,000 cells/mL, microcystin toxin ≥20 µg/L or visible cyanobacteria surface accumulation. The water body would be re-tested within one week. The Warning remained in effect until the cyanobacteria concentrations were <100,000 cyanobacteria cells/mL and the microcystin toxins <20 µg/L for two consecutive weeks at all sample sites. Bodies of water that fell below these levels would still be within an Advisory level and remain on a public health alert.

The Kansas Department of Health and Environment updated the Harmful Algal Bloom Policy and Response plan in April, 2012. A public health Advisory was issued when microcystin toxin levels were ≥4 to <20 µg/L; the other conditions that required issuing a public health alert remained the same.

### 3.2. Outreach to Physicians and Veterinarians

A letter was sent to healthcare providers and veterinarians to alert them of the signs and symptoms of FHAB-related health events and to encourage them to report cases to KDHE. A letter, written by the KDHE Secretary of Health, was mailed to members of the Kansas Academy of Family Physicians on May 23, 2011. This letter was also distributed electronically through the Kansas Health Alert Network. An article written by the State Public Health Veterinarian was distributed to veterinarians via e-mail to members of the Kansas Veterinary Medical Association on July 20, 2011.

**Table 5:** CDC proposed case definitions for algal toxin-related diseases. FHAB, freshwater harmful algal bloom.

Case	Suspect	Probable	Confirmed
**Animal**	Exposure to water or to seafood with a confirmed algal bloom AND onset of associated signs within a reasonable time after exposure AND without identification of another cause of illness.	Meets criteria for *Suspect Case* AND there is laboratory documentation of HAB toxin(s) in the water.	Meets criteria for a *Probable Case* combined with professional judgment based on medical review. or Meets criteria for a *Probable Case* and documentation of a HAB toxin(s) in a clinical specimen, provided appropriate testing is available.
**Human**	Same as animal *Suspect Case*.	Same as animal *Probable Case*.	Meets criteria for a *Probable Case* combined with professional judgment based on medical review.

### 3.3. Case Reports and Investigations

FHAB-related illness reports were made by phone or through an online reporting system available on the Kansas Department of Health and Environment website. Each report was reviewed by an epidemiologist, and KDHE Environmental Field Services staff collected water samples for testing. The Centers for Disease Control and Prevention’s (CDC) proposed case definitions for algal toxin-related diseases were used to classify animal and human cases (Table 5). Suspect, probable and confirmed human and animal cases were submitted to the Harmful Algal Bloom-related Illness Surveillance System (HABISS) of the National Center for Environmental Health (NCEH), Centers for Disease Control and Prevention. 

## 4. Discussion

In 2011, all confirmed FHAB-associated illnesses and deaths in dogs and 70% (5/7) of confirmed FHAB-associated illnesses in humans were exposed at Milford Lake. KDHE sampled Milford Lake on July 20, 2011. All animal and human cases (Cases 2, 3, 5, 6 and 7) were exposed at Milford Lake between July 20 and September 24. Milford Lake has an estimated 500,000 visitors each year [12]. The majority of these visits likely included some form of recreational water use that placed people, and animals, at risk for exposure to the harmful algal bloom. Due to the high cyanobacteria cell concentrations, level of microcystin and duration of the FHAB, it is surprising that there were not more cases of FHAB-related illnesses reported. Shoreline areas were closed, and there were intensive outreach efforts to educate the general public on FHABs through weekly media releases, website updates, printed brochures and informational signs posted at the lake. Despite these efforts, four confirmed human cases of FHAB-associated illness and all dog deaths occurred after a public health alert was issued for Milford Lake. There are many people who visit from outside the local area and may not be familiar with the appearance of a FHAB or local news media stations that would report public health alert messages. We recommend all FHAB-related case investigations include questions on the knowledge of FHABs and public health alerts, as applicable. This will allow public health officials to determine the effectiveness of messaging and to determine the best use of limited resources for education and outreach to prevent FHAB-associated morbidity and mortality.

The demographic data from our cases differ from national freshwater FHAB-associated outbreak cases. From 2009 to 2010, 11 waterborne disease outbreaks associated with freshwater algal blooms were reported to CDC [8]. These outbreaks caused at least 61 illnesses and two hospitalizations [8]. Demographic information was available for 34 ill persons; 38 (66%) were aged ≤19 years of age. The median age of confirmed human cases during our study period was 40 years; there was only one pediatric case reported (Case 2). If we expand our case-patient analysis to include suspect (*n* = 1) and probable (*n* = 5) cases, there were only two additional FHAB-related illnesses in children (two years old and 19 years old) This is surprising, as children are more sensitive to FHAB toxins than adults and have a penchant for risky behaviors, such as drinking lake water [7].

FHAB-related illness can be mild and self-limiting; however, the spectrum of illness can be severe. We report the first detailed information of two hospitalized case patients with recreational exposure to a confirmed freshwater harmful algal bloom in the United States. FHAB-related illness is a diagnosis of exclusion; although microcystin toxin can be identified in biological specimens (e.g., blood, vomitus), these tests are not readily available. A healthcare provider may find it difficult to confirm that FHAB toxins are the cause of a patient’s illness based on symptoms alone [7]. The hospitalized case patients relayed information on their exposure to a FHAB during the initial consultation with their healthcare provider. An accurate exposure history provided by the case patient, the healthcare provider’s ability to rule-out other likely causes of the patient’s illness and the healthcare provider’s report of the cases to public health authorities were crucial components to confirm a FHAB-related health event. Each report of human or animal illness potentially associated with a FHAB should be reported to public health authorities. An investigation of the implicated water body and subsequent testing for cyanobacteria and toxins is needed to confirm the presence of a FHAB and to classify a patient as a case. Case reports and epidemiological information on FHAB outbreaks should be published to add to the knowledge of this emerging public health threat. Reports of FHAB-associated outbreaks can be made through the National Outbreak Reporting System (NORS), as the Harmful Algal Bloom-Related Illness Surveillance System (HABISS) has been discontinued [8].

We collected five water samples weekly at Milford Lake and monitored cyanobacteria cell concentration and microcystin toxin levels. Variations in wind speed and direction, in part, may account for variations in cyanobacteria cell concentrations and microcystin at the five sample locations in Milford Lake [16]. Animal behavior may also contribute to risk, particularly in dogs that are prone to exploring shorelines and may seek out and ingest small pockets of accumulated algal scum that are present along the shoreline, but are not apparent from water test results. In addition, there was a delay of up to several days between exposure and when the case patient was reported to public health authorities. Ideally, water samples should be collected at the time and location of exposure.

Two human case patients reported exposure to Milford Lake in 2012, and one dog was reported to be exposed at Milford Lake in 2013. Milford Lake was under a public health alert for a FHAB for five weeks during 2012 and four weeks in 2013. The reduction in case reports is likely due to at least two factors. First, although Milford Lake experienced a FHAB in both 2012 and 2013, the duration of the FHAB was significantly shorter, reducing the amount of time humans, and animals, could be exposed. Second, an aggressive education and outreach effort by FHAB partners, including the media, to recreational water users, healthcare providers and veterinarians likely contributed to the reduction in case reports. However, an evaluation of FHAB public health messaging to recreational water users is needed to validate and quantify this hypothesis.

This study is subject to at least two limitations. First, under-reporting of human and animal cases to public health authorities may have occurred. Human healthcare providers and veterinarians must first recognize that a patient’s symptoms or clinical signs may be due to exposure to a FHAB, rule-out other likely causes of illness and then report the case to public health authorities. KDHE requests that human healthcare providers and veterinarians report FHAB-related cases; however, it is not mandatory. Second, case misclassification of FHAB-related cases may have occurred. The majority of human cases classified as “not a case” were due to the absence of laboratory testing for other etiologies that may explain a patient’s symptoms.

No U.S. Federal policy, regulations or guidelines exist for FHABs in recreational waters; however, several states have developed their own policies [1,9,10]. The state of Nebraska demonstrated a robust public health response to reports of two dog deaths from cyanobacterial toxicosis in 2004 [9]. The implicated lake was sampled for total microcystins; levels exceeded 15 μg/L, and a health alert was issued. Due to the short amount of time between the alert and the weekend, when the lake was heavily patronized, numerous people were exposed, and more than 50 cyanobacteria health-related complaints were documented. Currently, the Nebraska Department of Environmental Quality conducts weekly or bi-weekly sampling at 47 public lakes from May through September, and results are updated weekly on their website (http://deq.ne.gov/NDEQProg.nsf/Beaches2014.xsp). A lake is placed on a public health alert if the microcystin concentration is ≥20 μg/L.

## 5. Conclusions

The incidence of freshwater harmful algal blooms has increased in Kansas and caused human illnesses, including two hospitalized case patients and several dog deaths. The Kansas Department of Health and Environment, in conjunction with their local and national partners, developed a Harmful Algal Bloom Policy and Response Plan based on historical lake water sample data and incorporated human and veterinary case reports as a part of its core surveillance activities. Voluntary reports of FHAB-related cases by human and veterinary healthcare providers and investigation of each report by public health officials were critical components to the FHAB surveillance system. The annual review of the water sample data and human and veterinary surveillance data guided changes to the policy and response plan.

The Kansas experience, as described in this article, demonstrates the importance of a systematic data collection system to document the impact of FHABs on human and animal health. States without a formal FHAB program should consider the development of a policy to address this emerging issue to prevent morbidity and mortality, among humans and animals, at public recreational water venues. In addition, public health officials and FHAB partners must ensure continued awareness of the risks to the public, educate healthcare providers and veterinarians on FHAB-related illness and encourage timely reporting to public health authorities each year.
